# Medical Chemistry

**Published:** 1879-10

**Authors:** 


*Medical Chemistry*, including the outlines of Organic and Physiological Chemistry. By C. Gilbert Wheeler, Professor of Chemistry in the University of Chicago, etc. Publishers: Lindsay & Blakiston, Philadelphia, 1879.

This work is designed for the medical and dental students, and is of a size suitable for a text-book, and so arranged as far as convenience in method and form of expression are concerned, as to make it a comprehensive and useful treatise.

The author assumes that the study of inorganic chemistry has previously engaged the attention of students before entering college, or at least that they have become familiar with the general principles of modern chemical philosophy, and hence a estatement of the theory of chemistry is omitted.

Riche's, Manuel de Chimie, forms, in part, the basis of the outlines on Organic Chemistry, and the latest authors have been consulted in Prof. Wheeler's work. The centigrade thermometer and the metric system of weights and measures are generally employed throughout this treatise, and the dental tissues receive due notice. The style of the work reflects credit upon those veteran publishers of medical and dental literature, Messrs. Lindsay and Blakiston, and no doubt this effort to present a standard text-book in such a concise and perspicuous form will be duly appreciated.

